# Cost of a 5-year lung cancer survivor

**DOI:** 10.1038/sj.bjc.6605254

**Published:** 2009-09-08

**Authors:** S W Duffy

**Affiliations:** 1 Centre for Maths, Stats and Epidemiology, Cancer Research UK, Wolfson Institute of Preventive Medicine, Charterhouse Square, London EC1M 6BQ, UK

In their paper, Castleberry *et al* (2009) propose an economic approach, which estimates cost-effectiveness in terms of the cost per 5-year survivor instead of per year of life gained. This is an interesting approach in oncology, in which clinical results are very often presented as 5-year survival rates. It also raises interesting issues. For example, the inbuilt advantage to preventing deaths earlier in life when using the cost per year of life saved will be less pronounced with the proposed measure. The methodology of estimation is rigorous and painstaking. The controversial aspect of Castleberry *et al*'s work is the application of their approach to CT screening for lung cancer. The survival of screen-detected cases is known to be potentially affected by self-selection for screening, lead-time bias, length bias, and its extreme form, overdiagnosis.

It is the last potential bias that is probably the most important issue in Castleberry *et al*'s evaluation of lung cancer screening. A long-term follow-up of the Mayo Lung Project suggests that there is an element of overdiagnosis in conventional chest X-ray screening for lung cancer (Marcus *et al*, 2006). Owing to its greater sensitivity, it is difficult to avoid the conclusion that there must be some overdiagnosis in CT screening.

The time to death from lung cancer in patients with the disease is likely to be a mixture of continuous distributions, as is the time to death from other causes. It is inevitable that in some cases the latter occurs before the former, and that in some screen-detected cases the latter will occur *before* the date on which the disease would have been symptomatically diagnosed. This brings us to the observation that overdiagnosis has an epidemiological rather than pathological definition. It is defined as the diagnosis by screening of a disease that would never have been clinically diagnosed in the lifetime of the host had screening not taken place.

The above definition does not refer to the pathological attributes of a tumour, but intuitively, one would expect overdiagnosed cases of any cancer to be largely characterised by favourable stage, type, and other pathological factors. The discussion of overdiagnosis in the context of lung cancer has tended to focus on the suspicion that there is a substantial proportion of screen-detectable cancers that have little or no potential to cause symptoms or threaten life (Dammas *et al*, 2001).

A number of randomised trials of CT screening for lung cancer are in progress, and there is a possibility of these being augmented by a trial in the UK. Only such randomised trials will be able to establish the existence of a mortality benefit and, if relevant, estimate its size. They will also help to inform us about the existence and magnitude of overdiagnosis. One aspect of this that has been neglected is that if the hypothesis of a sizeable population of indolent lung tumours is correct, this is a fascinating phenomenon, given the very poor prognosis of symptomatic disease. If such a population exists, the challenges will be to find pathological or biological markers to identify it, and to research interventions that will make the remaining lung tumour population behave in the same benign manner.

The primary purpose of the CT screening trials is of course to inform us of its efficacy. In the absence of such trial results, any cost-effectiveness estimates are inevitably speculative, assuming a life year's benefit that has not yet been proven. Preliminary estimates may, however, be of some use in planning till the time the trial results are available. However, such estimation should take account of the important potential biases, for which methods are available (Duffy *et al*, 2008). Further arithmetic adjustment in addition to the analysis of Castleberry *et al* may seem to be a daunting prospect. For credible results, however, addressing the potential biases is absolutely necessary.
